# Oral Health-Related Quality of Life in Temporomandibular Disorder Patients and Healthy Subjects—A Systematic Review and Meta-Analysis

**DOI:** 10.3390/diagnostics14192183

**Published:** 2024-09-30

**Authors:** Lujain AlSahman, Hamad AlBagieh, Roba AlSahman

**Affiliations:** 1 Oral Medicine and Diagnostic Sciences Department, College of Dentistry, King Saud University Riyadh, Riyadh 57448, Saudi Arabia; 2 Faculty of Dentistry, Royal College of Surgeons, D02 YN77 Dublin, Ireland

**Keywords:** oral health-related quality of life, temporomandibular disorders, meta-analysis, GRADE analysis

## Abstract

(1) Background: Temporomandibular disorders (TMD) signs and symptoms affect the quality of life of patients because they impose an incapacity to participate in daily life activities, causing both physical and psychological discomfort. This review aims to provide the most accurate, comprehensive, and up-to-date description of all information available regarding OHRQoL in TMD. (2) Methods: A systematic search of articles from January 2013 till August 2023 was performed on five databases to identify articles, including TMD and oral health-related quality of life. Two calibrated reviewers performed the search following inclusion and exclusion criteria. A manual search of reference articles was also performed. The data were analyzed qualitatively by combining a meta-analysis and GRADE evidence. The Newcastle–Ottawa scale for cross-sectional and case-control studies was utilized to assess the quality of the included studies. (3) Results: The initial search consisted of 738 articles without the removal of duplicates. Fifteen articles were included in this systematic review, and ten were included in the meta-analysis. Almost all the included observational studies reported poor OHRQoL among patients with different types of TMD. The results of the meta-analysis with a standard mean difference (SMD) and that included seven studies suggest high heterogeneity with I^2^ = 99%, SMD (95% CI) = 3.18 (1.90, 4.46), *p*-value < 0.01. The odds ratio analyzed for three included articles in the meta-analysis reported statistical significance (*p*-value < 0.01) with OR = 8.21 (2.39, 28.25) and a heterogeneity of 86%. The certainty of evidence by GRADE resulted in a downgraded level of evidence, indicating that the OHRQoL of TMD patients may differ slightly from the healthy controls. (4) Conclusions: The impact of OHRQoL on the TMD was deemed to be significant. Overall, the OHRQoL is low for any type and intensity of pain among TMD patients and controls.

## 1. Introduction

Temporomandibular disorders (TMD) is a group of musculoskeletal disorders that affect the masticatory muscles, the temporomandibular joint (TMJ), and the related structure according to the American academy of orofacial pain [1]. The common signs and symptoms of TMD include muscular and/or articular pain, joint stiffness, clicking, asymmetric joint movements, and limited mouth opening. Additionally, patients with sleep disorders are more predisposed to TMD signs and symptoms, increasing the psychological issues of the patients [2,3]. Due to physical and psychological discomfort, patients with TMDs show poor oral health-related quality of life [4]. The World Health Organization (WHO) defined oral health-related quality of life (OHRQoL) as an individual’s perception of oral health and how it impacts their overall well-being, daily functioning, and quality of life [5]. Measuring OHRQoL is not limited to diagnosing oral diseases and considers the perspective of a person on their subjective experience, including their ability to eat, speak, and socialize without discomfort or embarrassment [6]. Previous models on oral health-related quality of life were directly associated with oral conditions and quality of life [7]. While the present models are designed to connect a person’s mind and overall health as a single unit and concentrate on socioenvironmental factors along with oral diseases. OHRQoL questionnaires consider how oral health conditions can affect a person’s physical, psychological, and social well-being [8]. This concept recognizes that oral health is an integral part of general health and that improving oral health can positively impact a person’s overall quality of life. This approach ultimately helps policymakers and investors plan strategies in favor of oral health and improving the well-being of an individual. In an observational study by Almoznino et al., reduced OHRQoL was reported in patients with TMD compared to healthy adults [9]. Similarly, in a systematic review of clinical studies by Dahlstrom and Carlsson (2010), all included studies measured reduced general quality of life in patients with TMD than the controls [10]. The advancement of research that focuses on the relationship between oral health-related quality of life and various prevalent oral diseases is crucial for effectively allocating public and private financial companies to address urgent and impactful healthcare needs guided by the principle of equitable care [11]. Recent studies have explored the association between temporomandibular disorders (TMD) and quality of life, but these studies exhibit varying methodologies and reported diverse outcomes concerning both quality of life and a TMD diagnosis [10,12,13,14]. A systematic review indicated that TMD patients experience a lower quality of life than non-TMD individuals [15]. However, this review did not provide specific data for TMD diagnostic groups categorized under the Research Diagnostic Criteria for Temporomandibular Disorders (RDC/TMD) axis I, such as muscle disorders (group I), disc displacements (group II), and joint dysfunction (group III). Introduced in 1992, the RDC/TMD became a standard tool in TMD research until the development of the Diagnostic Criteria for Temporomandibular Disorders (DC/TMD), which, as an enhanced version, has since become the more reliable tool. The DC/TMD provides clinicians with evidence-based criteria for assessing patients and improving communication about consultations, referrals, and prognoses. The importance of these criteria relay on providing a comprehensive framework for assessing TMD, and both clinicians and researchers can identify and classify the type and severity of TMD accurately [16].

Moreover, none of these reviews also conduct a meta-analysis and GRADE evidence [15]. Moreover, all the reviews examined the overall quality of life and its association with TMD [10,15]. Therefore, this systematic review and meta-analysis with GRADE evidence focuses on the observational studies on TMD and oral health-related quality of life utilizing validated diagnostic tools. Hence, the aim of this prospective systematic review and meta-analysis is to compare the oral health-related quality of life between the patients with TMDs and healthy controls in observational settings that have utilized one of the diagnostic criteria (RDC/TMD and\or DC/TMD) and a valid questionnaire to measure OHRQoL. Moreover, the aim is to present the most up-to-date and comprehensive overview of all the data on TMD and oral health-related quality of life.

## 2. Materials and Methods

The present systematic review and meta-analysis followed the PRISMA guidelines (preferred systematic review and meta-analysis guideline) [17]. The research protocol was designed prior to the commencement of the study, and the protocol was registered in the PROSPERO database under registration number CRD42023417542.

### 2.1. Research Question

This review aimed to answer the research question: “Is there a difference in oral health-related quality of life in individuals with (Group 1 DC/TMD) compared to healthy adults?” The PECOT strategy followed was (P) 18–60-year-old patients with TMD; (E) diagnosis of TMD evaluated by RDC/TMD or DC/TMD; (C) individuals without any TMDs; (O) outcome measured with OHRQoL; and (T) observational studies (cross-sectional and case-control).

### 2.2. Inclusion and Exclusion Criteria

Inclusion criteria were as follows: (a) the study had to be observational (cross-sectional and case-control studies) and involve subjects aged 18 to 60 years, (b) the diagnostic criteria for temporomandibular disorders had to be the RDC/TMD or the DC/TMD (both axis I and axis II), and (c) standard questionnaires measuring the quality of life (OHIP-14 or OHIP-49) had to be used. The studies must have been published in peer-reviewed journals since January 2013 and be in English.

The exclusion criteria were as follows: (a) studies that did not focus on TMD and myofascial pain as the primary disease or outcome (b) studies involving patients who had undergone previous TMD treatments (such as oral splints, medication, joint replacement, etc.), (c) studies involving patients with a history of facial trauma or rheumatic diseases, and (d) studies that did not use standard research diagnostic questionnaires for TMD and/or quality of life assessments. Review articles, letters to editors, and interventional studies like RCTs were excluded.

### 2.3. Search Strategy

A systematic search was conducted on five databases, PubMed/MEDLINE, Cochrane, Embase, Scopus, and Web of Science, from January 2013 to August 2023. The database searches commenced on 5 October 2023. The title and abstract searches used general search terms with Medical Subject Headings (MeSH) from PubMed and MEDLINE. These terms and descriptors are listed in Table 1 alongside the articles extracted from various databases. Boolean operators ‘AND’ and ‘OR’ were employed to refine and broaden the search scope.

Additionally, two investigators conducted reference chasing and manual searches of articles. The reference lists in the bibliographies of the identified articles were also reviewed. Two investigators independently screened the titles and abstracts of all the papers from the initial search. Any disagreements between these two authors were resolved through the consensus of a third reviewer.

### 2.4. Data Extraction and Study Selection

The articles from the databases were organized using EndNote 21 (Thomson Reuters^®^, New York, NY, USA), and duplicates were removed. The article screening and selection process consisted of two stages, each conducted by two independent investigators: (a) the first stage involved reading and evaluating the titles and abstracts, and (b) the second stage involved reading the full-text articles and reaching a consensus. A third investigator was consulted in any disagreement, and their decision was considered final. The articles that were excluded during the full-text reading stage and the reasons for exclusion are listed in Table S1. For articles without the full text or that were missing information, attempts were made to contact the authors. In cases where no reply was obtained from the authors, the articles were purchased.

For data extraction, a customized Excel (Microsoft Office 365, Redmond, WA, USA) worksheet was created based on the “Cochrane Handbook for Systematic Review and Meta-analysis” following STROBE guidelines for cross-sectional and case-control studies by two investigators [18,19].

Based on recommendations following the guidelines, the information extracted from the articles were as follows: (1) Authors/Country/Year; (2) study design; (3) age range of participants and gender; (4) the number of patients and controls; (5) diagnostic criteria for TMD; (6) instrument utilized for measuring oral health-related quality of life; (7) data collection method; and (8) outcome of the study (comparison with control and effect on OHRQoL).

### 2.5. Risk of Bias and Data Analysis

The quality of articles included in this review was measured by the New Castle Ottawa (NOS) scale. This scale is based on the star system used to classify observational studies. A single star is assigned to measure the quality of specific items, providing a maximum score of seven. The NOS system for case-control study has three main domains: (a) selection of cases and control; (b) compatibility between the group; and (c) exposure and outcome variables of the study [20]. For cross-sectional studies, the major domains are (a) case selection (representativeness of cases, sample size, and surveillance tools), (b) compatibility, and (c) outcome (assessment of the outcome and statistical tests) [21]. Concerning data analysis, results were combined in a meta-analysis by graphical presentation with the forest plot. Only studies presenting sufficient data (sample of cases and control; mean and standard deviations for TMD patients and controls) were included. Articles without a control group that was not divided by the group of TMD were excluded from the meta-analysis. Ten articles were included for meta-analysis and I^2^ measured heterogeneity.

### 2.6. Certainty of Evidence (GRADE Analysis)

The evidence for comparison of included studies was evaluated with the grading of recommendation, assessment, development, and evaluation tool (GRADE tool, available online at gradepro.org). The GRADE evidence was assessed using each effect generated by comparison of all included studies. For all included studies, the certainty of the evidence was rated down if there was a problem due to the risk of bias, indirectness, inconsistency, imprecision, and publication bias. Also, evidence was rated if the study design was proper and the outcome measured was consistent [22].

## 3. Results

**Figure 1:**
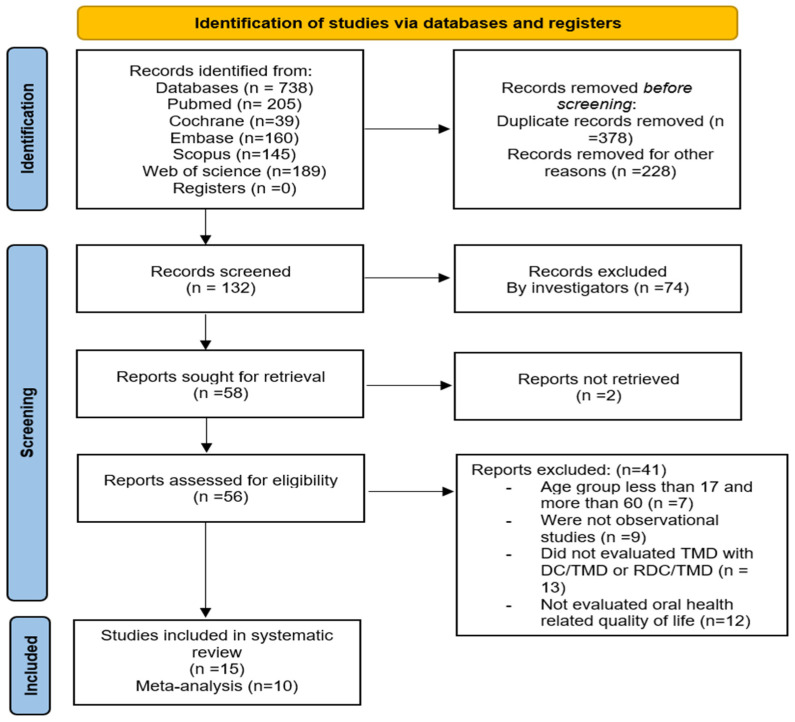
PRISMA flow chart for included studies.

A total of 738 studies were initially retrieved from various databases and synchronized in Endnote 21 (Clarivate, NY, USA). Among these, 378 studies were removed due to duplication and 228 due to other reasons (irrelevant study, letter to editors, etc.). The remaining 132 records underwent screening based on title and abstract, resulting in the removal of 74 studies (Figure 1). Subsequently, 58 articles were selected for full-text retrieval, but 2 could not be retrieved. Finally, 56 articles were considered for full-text reading, and 41 were excluded for various reasons (Table S1). Ultimately, 15 articles were included in this systematic review, with 10 were eligible for the meta-analysis.

### 3.1. Characteristics of Included Studies

Table 2 illustrates the summary and characteristics of the included 15 studies. Of all studies, eight were case-control [9,23,24,25,26,27,28,29], and seven were cross-sectional studies [2,30,31,32,33,34,35] reporting the OHRQoL of TMD patients; the oldest study was from 2013 [26,34]. A total of 4821 participants (cases 3945; control 1943) were included in this systematic review. All the included studies evaluated patients with TMD in hospital settings, and none were population based. The RDC/TMD axis I was applied in 11 studies [2,23,25,26,29,30,31,32,33,34,35], while DC/TMD with axes I and II were applied in 4 studies [9,24,28,36]. Four case-control studies utilized RDC/TMD analysis for all the cases [23,25,26,29], while three studies utilized DC/TMD [24,28,36], and one study utilized both RDC/TMD and DC/TMD for a diagnosis of TMD in the cases and controls [9]. Regarding the questionnaire measuring oral health-related quality of life, 13 studies utilized the oral health impact profile (OHIP)-14, and one study utilized OHIP-49 and the World Health Organization Quality of Life (WHOQOL) [34].

Based on Table 2, the included participants were in the age group of 18–60 years, confirming that the data from all the included studies were from young and middle-aged individuals from both genders. 

### 3.2. Oral Health-Related Quality of Life

All the included studies showed that TMD patients had worse oral health-related quality of life than healthy controls. Some studies have reported a direct relationship between the intensity of pain related to TMD and a worsened OHRQoL [28,34,35,36]. Coherently, two included articles on myofascial pain and TMD reported a worse OHRQoL of patients with severe pain compared to controls [34,35]. Additionally, two included cross-sectional studies have indicated that group I (with myofascial pain) and group III (with osteoarthritis) analyzed by RDC/TMD axis I had a worse OHRQoL compared to group II (with disc displacement) [33,34].

### 3.3. Result from Meta-Analysis

**Figure 2:**
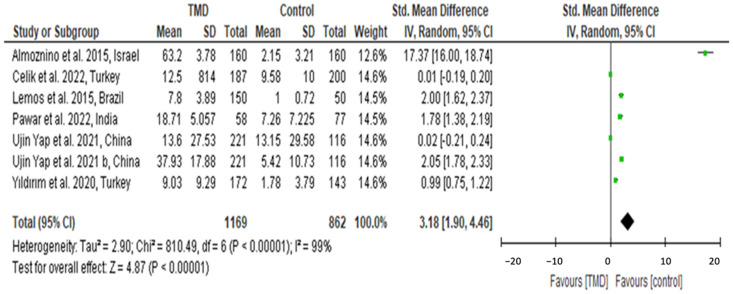
Forest plot for calculating standarized mean for OHRQoL among TMD patients and controls [9,24,27,29,32,33,37]. *The green dots represent the **standardized mean differences (SMD)** for each study, with horizontal lines indicating the **95% confidence intervals (CI)**. Values left of the vertical line favor the TMD group, while those to the right favor the control group.*

**Figure 3:**
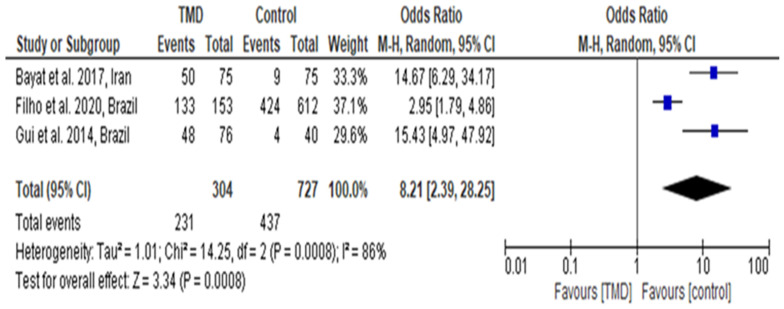
Forest plot for calculating odds ratio for OHRQoL among TMD patients and controls [23,25,31]. The blue dot represents the pooled standardized mean difference (SMD) with its 95% confidence interval (CI) for the overall meta-analysis.

Ten studies of fifteen were included in the meta-analysis due to high variability among the studies (Figure 2 and Figure 3). The included studies in the meta-analysis were the ones with an explicit inclusion of participants, standard deviation, and mean per group and compared OHRQoL between TMD and control groups. Studies included in this systematic review without proper comparison between TMD and controls were excluded from the meta-analysis. Two articles with the subgroups were not included as it was impossible to have accurate data for various groups.

Figure 2 included seven articles that reported oral health-related quality of life in global TMD patients and controls. All the included studies utilized RDC/TMD (with axis I or II) for diagnosis and OHIP-14 for evaluating OHRQoL.

The meta-analysis result reported high significance among the OHRQoL of TMD patients compared to the controls. The results are as follows: total participants: 2031 subjects; the standard mean difference (95% CI): 3.18 (1.90, 4.46); heterogeneity I^2^ 99% and Z test: 4.87, with the *p* < 0.01.

In Figure 3, the results of three studies have been pooled to calculate the odds ratio for measuring the difference in the OHRQoL of TMD patients and controls. The results indicated I^2^ = 86% and odds ratio = 8.21 (2.39, 28.25), which was found to be statistically significant (*p* < 0.05 and Z test = 3.34, respectively). Therefore, the findings of the meta-analysis indicate that patients with intense TMD pain had a worse OHRQoL compared to healthy adults.

### 3.4. Quality of Included Studies

Of the eight included case-control studies, four were of good quality, three were of moderate quality, and one was of poor quality on the NOS scale (Table 3). The studies lacked a proper diagnosis of TMD by utilizing RDC/TMD for controls, which caused hindrances in accurate diagnosis. Of the seven included in the cross-sectional study (Table 4), three were of good quality, two were of moderate and two poor quality on the NOS scale. The poor and moderate quality of the cross-sectional study was due to a comparison between the TMD group and control (most studies did not have controls) and an improper assessment of the findings.

### 3.5. GRADE Certainty of Evidence

The certainty of evidence among the included studies was low and downgraded, indicating imprecision, indirectness, inconsistency, and a high risk of bias (Table 5). The low level of the certainty of the evidence in this review indicates that the confidence in the effect estimate is limited, and the true effect may be substantially different from the estimate of the effect.

## 4. Discussion

This systematic review and meta-analysis aimed to compare the perception of oral health-related quality of life in patients with and without TMD. The included studies used RCD/TMD and/or DC/TMD and validated questionnaires for diagnosing the condition. Based on the analysis of this systematic review and meta-analysis with GRADE evidence, it was observed that oral health-related quality of life is worse for the patients suffering from TMD compared to the control group. Moreover, the patients diagnosed on RDC/TMD axis I with myofascial pain and osteoarthritis had a worse oral health-related quality of life than patients with disc displacement. Additionally, there was a direct relationship between the intensity of pain and a worse oral health-related quality of life.

Most articles included in this systematic review utilized RDC/TMD axis I for diagnosing TMD. However, RDC/TMD axis II has not been applied in several studies [24,27,36]. This is paramount when the study evaluates the OHRQoL, as axis II is used to diagnose the psychological factors associated with TMD (stress, anxiety, depression, and somatization). In the literature, it is mentioned that TMD pain not only worsens OHRQoL but also affects the psychological well-being of an individual [38]. Published systematic reviews and meta-analyses measured high depressive symptoms in patients with chronic TMD pain [10,39]. Oral health-related quality of life variables were evaluated by OHIP 14 and 49. These questionnaires are reliable and identify major aspects associated with OHRQoL [2,3,40]. One study has used the World Health Organization Quality of Life questionnaire, which mainly involves the general quality of life; however, one section of this questionnaire is mainly for evaluating OHRQoL [34]. Additionally, the WHOQOL also has a section involving the environment and the individual [34]. All the studies included in this analysis evaluated patients’ OHRQoL subscales, revealing that individuals with temporomandibular disorder (TMD) experience a compromised quality of life across all the subscales. Notably, pain emerged as the predominant factor affecting the quality of life in all subsets. Furthermore, the origin and etiology of TMD-related pain vary among individuals. Particularly, myofascial pain, associated with TMD, exhibits an intense pain of muscular origin [41]. Psychological complaints like depression and anxiety are common among patients suffering from TMD with muscular pain [14]. This is supported by the findings of an interventional study where patients receiving injections for TMD pain had a better quality of life compared to the placebo [42]. Additionally, the subscale of oral pain in patients with intervention was improved. Nowadays, researchers use a four-dimensional approach to evaluate the OHIP scale [43]. This approach includes oral pain, appearance, function, and psychological factors combined. Moreover, this approach provides a comprehensive result on oral health and its association with the intensity of pain [43]. Therefore, studies should report all seven subscales in OHIP to maintain the compatibility of the data provided.

In relation to meta-analyses, only ten studies were included due to high heterogeneity related to the methodological analysis, a lack of exposure, and the absence of the necessary data required for an analysis. However, it was observed that TMD negatively affects individual’s OHRQoL when compared to the non-TMD subjects. Additionally, OHRQoL was worsen in TMD patients in group I (myofascial pain) and group III (arthritis) compared to group II (disc displacement), with a statistically significant difference. These factors can be explained mainly by the worst pain levels in group I and III patients compared to group II, as reported in a systematic review on TMD pain and chronic pain analysis. Additionally, the presence of anxiety, depression, and somatization was reported worse in group I TMD patients compared to group II and III, also negatively impacting OHRQoL [44]. A systematic review on the therapeutic intervention of TMD reported improvement in pain intensity if the psychological factors along with pain medication are controlled in the patients with severe pain [45]. Most of the included studies measured that females with chronic TMD have two times worse OHRQoL than males. This difference could be attributed to the role of gender, considering that females are more likely to develop TMD-related pain and seek treatment faster compared to males. However, the literature suggests contradictory results when comparing the severity of pain and quality of life among genders, and only one study found functional limitations in females suffering from TMD-related pain [37]. Due to the heterogeneity among the included studies, a random effect model was used, and the study reported that the observed effect is an estimate of the real effect and follows the general distribution of the studies with smaller sample sizes gaining the weight compared to larger sample sizes. To improve future systematic reviews with a meta-analysis and to reduce the heterogeneity, it is suggested that the future systematic reviews should either apply the RDC/TMD or DC/TMD with axis I and II and comparing the overall quality of life measures in observational studies with larger sample size. Additionally, studies should clearly report the sample size, the mean, median, and standard deviation for the RDC/TMD diagnosis with axis I, II, and III groups. The use of RDC/TMD axis II is important, as it demonstrates the psychological aspects of the patients suffering from TMD-related pain. These findings are important when considering a person’s OHRQoL. Finally, studies should also report the sample source, method of randomization, qualification of examiner, and inclusion and exclusion criteria for both cases, and controls should be provided to avoid the bias related to the selection of participants.

Based on this systematic review and meta-analysis, several recommendations could be made for future studies. Firstly, studies should include both axis I and II for TMD patients, as this could not only focus on the intensity of pain but also the psychological variables associated with the severity of pain. Future studies should also focus more on the general quality of life because of the various etiological factors associated with TMD. It is paramount that future studies be more focused on investigating cases and controls with either RDC/TMD or DC/TMD to avoid bias related to case selection. In addition, the role of gender should also be assessed to evaluate the difference in OHRQoL among the genders. Finally, more clinical studies are required that focus on the data collection process of TMD and quality of life among various subgroups of TMD.

In summary, this systematic review with a meta-analysis and GRADE evidence shows that the OHRQoL is directly related to chronic TMD. The disability associated with TMD pain is also mentioned in this review. Therefore, TMD with (disc displacement) have an acceptable OHRQoL compared to myofascial pain and arthritis patients.

### Strengths and Limitations

Three qualified researchers conducted the evaluation, and the present systematic review adhered to PRISMA guidelines. Despite adhering to PRISMA guidelines and conducting a thorough quality appraisal of all included studies to comprehensively assess the oral health-related quality of life (OHRQoL) in patients with temporomandibular disorders (TMD), there are certain limitations that need consideration.

First, many included studies lacked important information, and some studies only provided details on TMD patients without a specific comparison to healthy subjects.

This systematic review and meta-analysis may be influenced by selection and measurement biases. All the included studies were of an observational nature, which limits the ability to establish causal relationships between TMD and OHRQoL. The inclusion criteria that required studies to be published in English since January 2013 and to use particular diagnostic criteria and questionnaires might have led to the exclusion of relevant research, such as studies in other languages or those using different diagnostic approaches. Additionally, the variability in diagnostic criteria (RDC/TMD or DC/TMD) and OHRQoL measurement tools (e.g., OHIP-14, OHIP-49) across studies could affect the comparability of results and the consistency of findings.

Second, all the included studies were of an observational nature, which limits their ability to establish causal relationships between TMD and OHRQoL. Additionally, the heterogeneity in study designs and measurement tools used to assess OHRQoL may introduce variability in the results. Most of the studies did not present the results in the function of RDC or DC subgroups. Therefore, in future research, standardizing diagnostic criteria using DC/TMD would ensure consistency in the definition and classification of TMD across studies. Uniform study designs to minimize variability in research methodologies will allow for straightforward comparisons between studies and contribute to a more coherent body of evidence, thereby enhancing the generalizability of findings.

## 5. Conclusions

Based on the results of this systematic review and meta-analysis with GRADE evidence, it can be concluded that patients with temporomandibular disorders (TMD) experience a lower oral health-related quality of life (OHRQoL) compared to healthy adults. This decline in OHRQoL is directly associated with the intensity of pain and disability as reported in individuals diagnosed with RDC/TMD and DC/TMD with axis I and II. Furthermore, the analysis revealed that pain intensity was higher among groups with arthritis and myofascial pain in comparison to those with disc displacement and healthy individuals. To gain a more comprehensive understanding of the impact of TMD on patients’ quality of life, further investigations are required. These should include the assessments of general quality of life, OHRQoL questionnaires, population-based data, and the diagnosis of TMD patients and controls using validated recent methods, such as DC/TMD, for a more accurate analysis of the effects on the quality of life of TMD patients.

## Figures and Tables

**Table 1:** MesH terms utilized in various databases.

Databases Searched	MesH Terms	Articles Found
PubMed	“Temporomandibular disorders [MeSH Terms] OR Temporomandibular Joint Disorders OR TMD” [MesH Terms] OR “temporomandibular joint disorder” [All Fields] OR “temporomandibular joint disease” [All Fields] OR “temporomandibular joint diseases” [All Fields] OR “temporomandibular joint dysfunction syndrome” [MeSH Terms] OR “temporomandibular joint dysfunction syndrome” [All Fields] OR “temporomandibular joint syndromes” [All Fields] OR “tmj disease” [All Fields] OR “tmd” [All Fields] OR “tmj” [All Fields] OR “tmjd” [All Fields] OR “tmj disorders” [All Fields] OR “tmj disorder” [All Fields] OR “tmj diseases” [All Fields] AND “orofacial pain” [Mesh term] OR “craniofacial pain [MesH term]” AND “Oral health [MeSH Terms] OR Oral Health OR Oral Health-Related Quality of Life [All Fields] OR OHRQoL [All Fields] OR Oral Health-Related Quality of Life [Mesh Terms] OR OHRQoL [MesH terms]	Initial search 205 Abstract and Title 26
Scopus	TITLE-ABS-KEY (temporomandibular disorders OR temporomandibular joint disorders OR TMD) AND (oral health-related quality of life OR oral health OR OHRQoL) AND (orofacial pain OR craniofacial pain)	Initial search 145
Web of science	(TS = (“temporomandibular joint disorder” OR “temporomandibular joint disorders” OR “temporomandibular dysfunctions” OR “temporomandibular joint syndrome” OR “tmj disease” OR tmjd OR “tmj disorders” OR “tmj disorder” OR “tmj diseases” OR “temporomandibular joint dysfunction syndrome”) AND TI = (“oral health related quality of life” OR “OHRQoL”)) AND TI = (“orofacial pain” OR “craniofacial pain”) AND DOCUMENT TYPES: (Article)	Initial search 189
EMBASE	#1 = (‘temporomandibular joint disorders’:ta,ab OR ‘tmj disorder’:ta,ab OR ‘temporomandibular joint disease’:ta,ab ‘temporomandibular joint dysfunction syndrome’:ta,ab) #2 = (‘oral health related quality of life’:ta,ab OR ‘OHRQoL’:ta,ab) #3 = (‘orofacial Pain’ OR craniaofacial pain’) AND #4 = ([article]/lim) #5 = #1 AND #2 AND #3 AND #4	Total articles: 160
Cochrane	(TS = (“temporomandibular joint disorder” OR “temporomandibular joint disorders” OR “temporomandibular dysfunctions” OR “temporomandibular joint syndrome” OR “tmj disease” OR tmjd OR “tmj disorders” OR “tmj disorder” OR “tmj diseases” OR “temporomandibular joint dysfunction syndrome”) AND TI = (“oral health related quality of life” OR “OHRQoL”)) AND TI = (“orofacial pain” OR “craniofacial pain”) AND DOCUMENT TYPES: (Article)	Total articles:39 Abstract and title: 2

**Table 2:** Characteristics of included studies.

Study No.	Author/Year/Country	Study Design	Number of Participants (Cases and Control)	Age (AR) and Gender	Case Selection Method	Control Selection Method	Diagnostic Criteria for TMD	Measurement of Oral Heath-Related Quality of Life	Method for Data Collection	Results/Conclusion
1	Celik et al., 2022 [24] Turkey	Case-Control Study	*n* = 200 (Case = 150; Control = 50)	AR = 18–60 years (F = 121; M = 79)	Patient seeking treatment for TMD	Not specified	DC/TMD for all cases	OHIP-14	Questionnaire	OHIP score was higher in patients with disc displacement and TMD-related pain compared to healthy controls
2	Pawar et al., 2022 [33] India	Cross-sectional comparitive study	*n* = 320 (Cases = 160; Control = 160)	AR = 18–60 years (F = 168; M = 152)	Patient seeking treatment for TMD (divided in three groups; G1 = myofacial pain, G2 = disc displacement, G3 = osteoarthritis	Patient seeking dental treatment for other reasons	RDC/TMD axis I and II for all the cases	OHIP-14	Questionnaire	Poor OHRQoL was reported in TMD patients compared to healthy controls
3	Ujin Yap et al., 2021 [28] China	Case-Control Study	*n* = 961 (Cases = 816; Control = 147)	AR = 18–40 years (F = 761; M = 200)	Not specified	Not specified	DC/TMD-SQ axis I for all the cases	OHIP-14	Questionnaire	OHRQoL is affected by different types of TMD symptoms. Individuals having more and pain-related TMD symptoms with/without intra-articular features generally had greater OHRQoL impairments.
4	Ujin Yap et al., 2021 [36] China	Case-Control Study	*n* = 961 (Cases = 845; Control = 116)	AR = 18–40 years (F = 761; M = 200)	Patient visted oral and maxillofacial clinics	Patients visited in prosthetic dental clinics	DC/TMD axis II for all the cases	OHIP-14	Questionnaire	Correlations between TMD severity OHRQoL were moderately strong to strong (rs = 0.42–0. 72)
5	Filho et al., 2020 [25] Brazil	Case-Control Study	*n* = 765 (Cases = 153; Control = 612	AR = 18–25 years (F = 765)	Not specified	Not specified	RDC/TMD axis II for all the cases	OHIP-14	Questionnaire	Women with negative OHRQoL reports symptoms of TMD
6	Yıldırım et al., 2020 [29] Turkey	Case-Control Study	*n* = 315 (Cases = 172; Controls = 143)	AR = 18–60 years(F = 192; M = 123)	Not specified	Not specified	RDC/TMD axis I and II for all the cases	OHIP-14 questionnaire	Questionnaire	Bruxers with TMD had poorer OHRQoL than those without TMD
7	Bayat et al., 2017 [23] Iran	Case-Control Study	*n* = 150 (Cases = 75;control = 75)	AR = 30–50 years (F = 119; M = 31)	Patient seeking treatment at clinic	Patients coming for follow-up of any dental treatment	RDC/TMD axis I and II for all the cases	OHIP-14	Interview	The TMD group had a worse quality of life than controls. The prevalence and severity of OHIP was 6 and 2 times higher, respectively, in the TMD group.
8	Balik et al., 2017 [30] Turkey	Cross-sectional study	*n* = 104 (Case and Control = not specified)	AR = 32–59 years(F = 64; M = 40)	Not specified	Not specified	RDC/TMD axis I and II for all the cases	OHIP-14	Questionnaire	OHIP score was higher in patients with disc displacement and TMD-related pain
9	Su et al., 2016 [35] China	Cross-sectional	*n* = 541 (Case and Control = not specified)	AR = 25–48 years (M = 134; F = 407)	Patient seeking treatment at clinic	Not specified	RDC/TMD axis I for all the cases	OHIP-14	Interview	OHIP score was worst in patients with muscular pain.
10	Almoznino et al. 2015, [9] Israel	Case-Control Study	*n* = 387 (Cases = 187 and Controls = 200	AR = 20–30 years (F = 111; M = 76)	Patient seeking treatment at clinic	Patient seeking treatment at clinic	DC/TMD and RDC/ TMDaxis I in cases	OHIP-14	Interview	TMD group showed statistical differences for the OHIP as compared to controls.
11	Lemos et al., 2015 [32] Brazil	Cross-sectional	*n* = 135 (Case and Control = not specified)	AR = 18–25 years (F = 77; M = 58)	Dental students	Not specified	RDC/TMD axes I	OHIP-14	Questionnaire	Severity of TMD impaired the oral health-related quality of life
12	Blanco-Aguilera et al. 2014, [2] Spain	Cross-sectional	*n* = 407 (Case and Control = not specified)	AR = 18–60 years (F = 365; M = 42)	Sample of the population of the Public Health System	Not specified	RDC/TMD (did not report axis)	OHIP-14	Interview	OHIP showed a significant and positive association in patients with painful TMD
13	Gui et al., 2014 [31] Brazil	Cross-sectional	*n* = 116 (Case = 76 and control = 40)	AR = 25–50 years (F = 116)	Sample from hospital records	Sample from hospital records	RDC/TMD for all the cases	OHIP-14	Questionnaire	Quality of life is significantly impared by widespread TMD pain compared to healthy control
14	Rener-Sitar et al. 2014, [26] Slovenia	Case-Control Study	*n* = 481 (Cases = 81 and Control = 400)	AR = 18–60 years (F = 365; M = 115)	Patients seeking treatment at the dental clinic	Random patients without TMD taking treatment at hospital	RDC/TMD axis I in all the cases	OHIP-49	Interview	TMD patients are highly associated with a lower OHRQoL.
15	Resende et al. 2013, [34] Brazil	Cross-Sectional	*n* = 43 (Cases = 43 divided in 3 groups G1 = 30; G2 = 9; G3 = 4)	AR = 30–45 years (F = 32; M = 11)	Patients seeking treatment at the dental clinic	Patients seeking treatment at the dental clinic	RDC-TMD axis I for cases	WHOQOL-BREF	Interview	Impiared oral health-related quality of life is recorded in patient with myofacial pain associated with TMD.

N = number of subjects; DC/TMD = diagnostic criteria for temporomandibular disorder; RDC/TMD =research diagnostic criteria for temporomandibular disorder; OHIP-14 = oral health impact factor-14; OHIP-49 = oral health impact factor-49, WHOQOL-BREF = World Health Organization quality of life-BREF.

**Table 3:** Risk of bias using NOS tool for case-control studies [20].

Studies		Celik et al., 2022 [24] Turkey	Ujin Yap et al., 2021 [28] China	Ujin Yap et al., 2021 [36] China	Filho et al., 2020 [25] Brazil	Yıldırım et al., 2020 [29] Turkey	Bayat et al., 2017 [23] Iran	Almoznino et al., 2015 [9] Israel	Rener-Sitar et al., 2014 [26] Slovenia
Selection	Case definition adequate	*	*	*	*	*	*	*	*
	Representativeness of cases	*	*	*	*	*	*	*	*
	Selection of controls	*	*	*	*	*	*	*	*
	Definition of controls	*	_	*	_	*	*	_	*
Comparability		**	**	**	*	**	**	**	**
Exposure	Ascertainment of exposure	_	*	*	_	*	_	_	*
	Same method of ascertainment for case and control	_	_	_	_	_	_	_	_
	Nonresponse rate	_	*	*	*	_	_	*	*
Quality		Moderate	Good	Good	Poor	Good	Moderate	Moderate	Good

NOS tool for cross sectional study is represented by * where; 1. **Representativeness of the sample**: a. Truly representative of the average in the target population. * 2. **Sample size**: a. Justified and satisfactory (≥100 patients included). * 3. **Non-respondents**: a. The response rate is satisfactory (≥90% of patients have anti-Ro levels available). * 4. **Ascertainment of the exposure (risk factor)**: a. Validated measurement tool used and anti-Ro52 has been distinctly measured. ** b. Validated measurement tool used but no distinction is made between anti-SSA/Ro and anti-Ro52. * **comparability**: (Maximum 1 stars) (a) The study investigates potential confounders. * Outcome: (Maximum 3 stars) (1) **Assessment of the outcome**: (a) Independent blind assessment. ** (b) Record linkage. ** (c) Self report. * (2) **Statistical test**: (a) The statistical test used to analyze the data is clearly described and appropriate *.

**Table 4:** Risk of bias using NOS tool for cross-sectional study [21].

Studies		Pawar et al., 2022 [33] India	Balik et al., 2017 [30] Turkey	Su et al., 2016 [35] China	Lemos et al., 2015 [32] Brazil	Blanco-Aguilera et al., 2014 [2] Spain	Gui et al., 2014 [31] Brazil	Resende et al., 2013 [34] Brazil
Selection	Representativeness of sample	*	*	*	*	*	*	*
	Sample size	*	*	*	*	*	*	*
	Nonrespondent	_	_	*	*	*	*	*
	Uncertainty of exposure	_	_	_	_	_	*	*
Comparability		**	_	*	_	_	**	**
Outcome	Assessment of outcome	*	*	*	*	*	*	*
	Statistical tests	*	*	*	*	*	*	*
Quality		Moderate	Poor	Good	Moderate	Poor	Good	Good

The NOS tool is represented by stars (*) as follows: **Representativeness of the Sample:** (*): Truly representative of the target population (all subjects or random sampling). (*): Somewhat representative of the target group (non-random sampling). (0 stars): Selected group of users/convenience sample. (0 stars): No description of the derivation of included subjects. **Sample Size:** (*): Justified and satisfactory (≥100 patients included). (0 stars): Not justified (<100 patients included). **Non-respondents:** (*): Response rate is satisfactory (≥90% of patients have anti-Ro levels available). (0 stars): Response rate is unsatisfactory (<90% of patients have anti-Ro levels available). **Ascertainment of the Exposure (Risk Factor):** (**): Validated measurement tool used and anti-Ro52 distinctly measured. (*): Validated measurement tool used but no distinction made between anti-SSA/Ro and anti-Ro52. (0 stars): Measurement methods not described. **Comparability:** (Maximum 1 star) (*): The study investigates potential confounders through subgroup analysis or multivariable analysis. (0 stars): The study does not investigate potential confounders. **Outcome:** (Maximum 3 stars) **Assessment of the Outcome:** (**): Independent blind assessment. (**): Record linkage. (*): Self-report. (0 stars): No description. **Statistical Test:** (*): The statistical test used is clearly described and appropriate. (0 stars): The statistical test is not appropriate, not described, or incomplete.

**Table 5:** Certainty of evidence (GRADE) (https://gdt.gradepro.org/app/#project) (accessed on 5 October 2023).

Outcomes	№ of Participants (Studies) Follow-Up	Certainty of the Evidence (GRADE)	Relative Effect (95% CI)	Anticipated Absolute Effects	
				Risk with Healthy Control	Risk Difference with Temporomandibular Disorder Patients
TMD and Myofascial pain and oral health-related quality of life (TMD) assessed with: Relative risk timing of exposure: mean 6 months	3945 cases 1943 controls (7 observational studies)	Low ^a,b^	RR 3.18 (1.90 to 4.46)	Low	
				520 per 1000	1134 more per 1000 (468 more to 1799 more)
TMD and Myofascial pain and oral health-related quality of life assessed with odds ratio follow-up: mean 6 months	304 cases 727 controls/exposed 304/991 unexposed (3 observational studies)	Low	OR 8.21 (2.39 to 28.25)	Study population	
				307 per 1000	477 more per 1000 (358 more to 619 more)
				Low	
				307 per 1000	477 more per 1000 (358 more to 619 more)

**The risk in the intervention group** (and its 95% confidence interval) is based on the assumed risk in the comparison group and the **relative effect** of the intervention (and its 95% CI). **CI:** confidence interval; **OR:** odds ratio; **RR:** risk ratio. Moderate certainty: moderately confident in the effect estimate: the true effect is likely to be close to the estimate of the effect, but there is a possibility that it is substantially different. Low certainty: confidence in the effect estimate is limited: the true effect may be substantially different from the estimate of the effect. Very low certainty: very little confidence in the effect estimates: the true effect is likely to be substantially different from the estimate of effect. ^a^: inconsistency in findings due to high heterogeneity related to study designs and recruitment of participants. ^b^: impression in findings was high due to high effect size and lower confidence interval.

## Data Availability

The data that support the findings of this study are available on request from the corresponding author.

## Supplementary Materials

The following supporting information can be downloaded at https://www.mdpi.com/article/10.3390/diagnostics14192183/s1, Table S1: Excluded studies for TMD-OHRQoL.
