# Supplementary material for: Oral Health-Related Quality of Life in Temporomandibular Disorder Patients and Healthy Subjects—A Systematic Review and Meta-Analysis
Source: Diagnostics (Basel). 2024 Sep 30;14(19):2183. doi: 10.3390/diagnostics14192183 (PMC11475636; doi:10.3390/diagnostics14192183)
Supplement: Supplementary file 1 [file diagnostics-14-02183-s001.zip › diagnostics-3153992-supplementary.pdf]

Sr.No	Authors/Year/Country	Title	Reason
1.	Linsen S.S et al., 2023, England	Is the risk of chronic pain after total temporomandibular joint replacement independent of its indications? A prospective cohort study	After treatment study
2.	Aktaş A. et al., 2023, Turkey	Dietary intakes of individuals with temporomandibular disorders: A comparative study	Dietary intake in TMD patients
3.	Yap AU et al., 2022, Singapore	Temporomandibular disorder symptoms in young adults: Three-dimensional impact on oral health-related quality of life.	Age ranges from 7–76 years
4.	Turcio KH et al., 2022, Brazil	Relationship of bruxism with oral health-related quality of life and facial muscle pain in dentate individuals.	Study on edentulous
5.	Tanner J et al., 2022, Finland	Health-related quality of life in patients with chronic orofacial pain compared with other chronic pain patients.	Other chronic pain
6.	Kroese JM et al., 2022, Amsterdam	Oral health-related quality of life in patients with early rheumatoid arthritis is associated with periodontal inflammation and painful temporomandibular disorders: a cross-sectional study.	Complete oral health
7.	Castaño-Joaqui et al., 2022, Brazil	Long term oral health related quality of life after TMJ arthrocentesis with hyaluronic acid. A retrospective cohort study	Treament outcomes
8.	Amaral-Freitas et al., 2021, Brazil	Impact of temporomandibular disorder on oral health-related quality of life in adolescents	Age ranges from 12 to 16 years
9.	Bahar et al., 2021, Malaysia	Temporomandibular disorder symptoms and their association with quality of life of dental patients in Malaysia	Age ranges from 13 to 78 years
10.	Lui et al., 2021, Japan	Temporomandibular disorder subtypes, emotional distress, impaired sleep, and oral health-related quality of life in Asian patients	Age ranges from 11 to 86 years
11.	Onada et al., 2021, China	Evaluation of oral health-related quality of life in patients with temporomandibular disorders	Before and after treatment OHRQoL was measured
12.	Brogårdh-Roth S, et al., 2021, Istanbul	Do preterm-born adolescents have a poorer oral health-related quality of life?	Complete oral health
13.	Oghli I et al., 2021, Sweden	The impact of oro-facial pain conditions on oral health-related quality of life: A systematic review.	Systematic review
14.	De Barros Pascoal AL, 2020, Brazil	Effectiveness of Counseling on Chronic Pain Management in Patients with Temporomandibular Disorders.	Calculating the effect of treatment
15.	Song YL and Yap AU, 2020, Singapore	Impact of pain-related temporomandibular disorders on jaw functional limitation, psychological distress and quality of life in postoperative class III East Asian	Comparison of class III malocclusion

		patients.	
16.	Kamaran et al., 2020, Turkey	Evaluation of temporomandibular disorders, quality of life, and oral habits among dentistry students	Oral habits
17.	Miller VE et al., 2019, USA	Characteristics Associated with High-Impact Pain in People With Temporomandibular Disorder: A Cross-Sectional Study.	No studied quality of life
18.	Su N et al., 2019, China	Prediction Models for Oral Health-Related Quality of Life in Patients with Temporomandibular Joint Osteoarthritis 1 and 6 Months After Arthrocentesis with Hyaluronic Acid Injections.	Treatment modalities
19.	Oberoi SS et al., 2019, India	Prevalence of Various Orofacial Pain Symptoms and Their Overall Impact on Quality of Life in a Tertiary Care Hospital in India.	Various treatment and their effect on OHRQoL
20.	Benoliel R et al., 2019, USA	Subjective Sleep Quality in Temporomandibular Disorder Patients and Association with Disease Characteristics and Oral Health-Related Quality of Life.	Overall oral health measures
21.	Natu et al., 2019, Singapore	Temporomandibular Disorder symptoms and their association with quality of life, emotional states and sleep quality in Southeast Asian youths	Age ranges from 15 to 65 years
22.	Hanisch M et al., 2019, USA	Oral symptoms and oral health-related quality of life of individuals with x-linked hypophosphatemia	Studied OHRQoL in TMd and hypophosphatemia
23.	Theroux J. et al., 2019, Spain	A cross-sectional study of the association between anxiety and temporomandibular disorder in Australian chiropractic students	Association between TMD and anxiety
24.	Wąsacz K.; Pac A.; Darczuk D.; and Chomyszyn-Gajewska M., 2019, Germany	Validation of a modified oral health impact profile scale (OHIP-14) in patients with oral mucosa lesions or periodontal disease;	OHRQoL in periodontitis patients
25.	Fan W.-Y. et al., 2019, Japan	Influence of clinical and psychological variables upon the oral health-related quality of life in patients with temporomandibular disorders	Studied various variables and their impact on OHRQoL
26.	Song Y.L.; Yap A.U.-J., 2018, Singapore	Outcomes of therapeutic TMD interventions on oral health related quality of life: A qualitative systematic review	Systematic review
27.	Da Silva F.C et al., 2018, Brazil	Impact of temporomandibular disorders and sleep bruxism on oral health-related quality of life of individuals with complete cleft lip and palate	Cleft lip–cleft palate patient OHRQoL
28.	Oghli I et al., 2017, Sweden	Prevalence and oral health-related quality of life of self-reported orofacial conditions in Sweden.	Complete oral health
29.	Lucas B. et al., 2017, Brazil	General health related quality of life in patients with TMD	General health

30.	Rahimi H et al., 2017, India	Orofacial symptoms and oral health-related quality of life in juvenile idiopathic arthritis: a two-year prospective observational study.	Age ranges from 12 to 18 years
31.	Benoliel R. et al., 2017, USA	Subjective sleep quality in temporomandibular disorder patients and association with disease characteristics and oral health-related quality of life	Sleep quality and other chronic pain
32.	Yamane-Takeuchi M.; et al., 2016, Japan	Associations among oral health-related quality of life, subjective symptoms, clinical status, and self-rated oral health in Japanese university students: A cross-sectional study	Self-reported pain and overall pain
33.	Barbosa T.S et al., 2016, Brazil	Factors associated with oral health-related quality of life in children and preadolescents: A cross-sectional study	Study on children and complete oral health
34.	Su N et al., 2016, Spain	Correlation between oral health-related quality of life and clinical dysfunction index in patients with temporomandibular joint osteoarthritis	Other index
35.	SILVOLA et al., 2015, Finland	Do changes in oral health-related quality-of-life, facial pain and temporomandibular disorders correlate after treatment of severe malocclusion?	Treatment of malocclusion
36.	Shueb S.S et al., 2015, England	What is the impact of acute and chronic orofacial pain on quality of life?	Evaluated quality of life for every type of oral and general pain
37.	Da Silva MF et al., 2014, Brazil	Temporomandibular disorders and quality of life among 12-year-old schoolchildren.	Age ranges from 7 to 12 years
38.	Blanco-Aguilera A. et al., 2014, Dutch	Application of an oral health-related quality of life questionnaire in primary care patients with orofacial pain and temporomandibular disorders	Not utilized RDC/TMD for TMD measurement
39.	Papagianni C.E, et al., 2013, Brazil	Oral health-related quality of life in patients with tooth wear	Not related to TMD
40.	MIETTINEN et al., 2012, Finland	Psychosocial aspects of temporomandibular disorders and oral health-related quality-of-life	Age range nor clear
41.	Tjakkes et al., 2010, Netherland	TMD pain: the effect on health-related quality of life and the influence of pain duration	Age from 16
